# Residents in difficulty—just slower learners? a case–control study

**DOI:** 10.1186/s12909-014-0276-z

**Published:** 2014-12-30

**Authors:** Lotte Dyhrberg O’Neill, Karen Norberg, Maria Thomsen, Rune Dall Jensen, Signe Gjedde Brøndt, Peder Charles, Lene Stouby Mortensen, Mette Krogh Christensen

**Affiliations:** Centre for Medical Education, INCUBA Science Park Skejby, Brendstrupgårdsvej 102, Building B, 8200 Århus N, Denmark; Postgraduate Medical Education in Region North, Skottenborg 26, 8800 Viborg, Denmark; Department of Internal Medicine, Randers Regional Hospital, Skovlyvej 1, 8930 Randers, Denmark

**Keywords:** Medical school performance, Postgraduate training, Problem residents, Residents in difficulty, Case–control study, Assessment

## Abstract

**Background:**

Recent meta-analyses have found small-moderate positive associations between general performance in medical school and postgraduate medical education. In addition, a couple of studies have found an association between poor performance in medical school and disciplinary action against practicing doctors. The aim of this study was to examine if a sample of Danish residents in difficulty tended to struggle already in medical school, and to determine whether administratively observable performance indicators in medical school could predict difficulties in residency.

**Methods:**

The study design was a cumulative incidence matched case–control study. The source population was all active specialist trainees, who were medical school graduates from Aarhus University, in 2010 to June 2013 in two Danish regions. Cases were doctors who decelerated, transferred, or dropped out of residency. Cases and controls were matched for graduation year. Medical school exam failures, grades, completion time, and academic dispensations as predictors of case status were examined with conditional logistic regression.

**Results:**

In total 89 cases and 343 controls were identified. The total number of medical school re-examinations and the time it took to complete medical school were significant individual predictors of subsequent difficulties (deceleration, transferral or dropout) in residency whereas average medical school grades were not.

**Conclusions:**

Residents in difficulty eventually reached similar competence levels as controls during medical school; however, they needed more exam attempts and longer time to complete their studies, and so seemed to be slower learners. A change from “fixed-length variable-outcome programmes” to “fixed-outcome variable-length programmes” has been proposed as a way of dealing with the fact that not all learners reach the same level of competence for all activities at exactly the same time. This study seems to support the logic of such an approach to these residents in difficulty.

## Background

The term “resident in difficulty” and many other synonymous terms have been used for medical residents who do not meet the expectations of the training programmes [1]. Residents in difficulty may suffer and also strain the learning environment and patient care [2]. It has been reported in the international literature that around 3–10% of doctors in postgraduate training are struggling to comply with educational requirements [1,3,4]. Early identification and support of residents in difficulty has been proposed as an important investment in the development and training of future health professionals, and as the gold standard for educational supervision [1,5-7]. The question is, if it is possible to identify risk factors during medical school before the unwanted outcome of a problematic residency manifests, so that resources may be directed towards preventive support of new residents at risk, instead of being used for damage control. At least three recent meta-analyses and one literature review examined the general association between performance in medical school and postgraduate medical education [8-11]. One meta-analysis including international studies reported a small effect size for previous academic performance as predictors of postgraduate performance [8]. In this study, the predictors of previous academic performance examined were solely grade-based and the outcome measure was mainly based on performance in the first year in training after qualification. A second meta-analysis based on international literature found undergraduate grades and rankings to be moderately correlated with internship and residency performance [9]. The third and most recent meta-analysis found performance measures available for US resident selection committees to be moderately, positively associated with resident performance [10]. Finally, a review of studies correlating mainly US medical student performances with subsequent residency performances also found positive correlations although the overall strength of the associations examined was unclear from this study [11]. In addition, a couple of studies have examined factors associated with extremely poor performances of practicing doctors [5,12,13]. Papadakis *et al*. found that the number of medical courses not passed during pre-graduate years 1–2, the MCAT z score, and reports of unprofessional behaviour in medical school were significant predictors of subsequent disciplinary action against practicing physicians [12]. British researchers also found failed exams in the early/preclinical courses in medical school to predict professional misconduct [5]. Generally, much of the existing research on residents in difficulty appears to be originating from English-speaking countries, in particular the US and the UK [8-11], a situation which may lead to systematic (location/regional) bias. No cohort study on the risk factors for being a resident in difficulty in a Danish context has previously been published nationally or internationally.

Danish residents are typically selected for specialist training positions based on their postgraduate curriculum vitae and on personal interviews. There is no tradition for using medical school performance indicators in the planning of residencies, i.e. residents are not evaluated on their profiles as learners in medical school, their habits or their learning needs in advance. Whether such an evaluation of medical school performance is justified at all in a Danish context has never been examined before, even though the aggregated international evidence as described above seem to generally suggest a small-moderate association between medical school performance and subsequent doctor performance. An important reason for the lack of validity studies for medical school performance in a Danish context is most likely that postgraduate medical performance is not graded with a score or a mark. The aim of this study was to examine *if* residents in difficulty in a Danish context tended to struggle already in medical school or not compared to controls, and to determine if/which administratively observable performance indicators in medical school would predict difficulties in residency. This study reports the results of a case–control study that examined medical school predictors of struggling to complete residency training as planned. The results will be discussed and compared with relevant literature in medical education.

## Methods

### Design

The study design was a cumulative incidence matched case–control study, which is rooted in epidemiological methodology, but the method is also used by the research community in medical education [5,12,14,15]. This design was chosen because residents in difficulty (cases) are relatively rare and because the time from exposure (early medical school) to manifest (struggling in residency) is long. The incidence of residents in difficulty has recently been estimated to be about 7% in the regions of our source population [16].

### Source population

The source population in this study was all active specialist trainees, who were graduates from Aarhus University, in 2010–June 2013 in two Danish regions (Central and North), which until recently was the only medical school in these regions. In other words, the source population constitutes medical doctors with at least two years of basic postgraduate clinical training who had begun their specialist training programmes. The general contract for specialist training is that residents are to complete their residency in four or five years depending on specialty to be authorized. Within this timeframe, they need to comply with a set of minimum competences, which are based on the CanMEDS physician competency framework [17], and complete a number of obligatory course and rotations in order to pass training. No grades are given in postgraduate training. The definition of residents in difficulty used by the postgraduate medical training authorities in our region was outlined in an official document entitled “Handling Problematic Residencies”. In this document a resident in difficulty is defined as “a doctor who does not acquire the required competences within the planned timeframe of the programme”. The three main strategies for dealing with these residents in the region are outlined in this document to be either: programme deceleration, ward transferral or even resident dismissal in the rare cases where problems remain unresolved and severe [18]. We therefore chose to define cases by a composite outcome, as doctors in residency who either: decelerated, transferred or dropped out. We included voluntary resignations and changes of speciality in the dropout category, because they are also indicative of problems with completing a programme as planned. We defined deceleration as having had long or repeated episodes of: leave of absence, unknown absence, illness, or delays due to inadequate development of competences. “Long” episodes of absence were defined as episodes lasting more than two months, and “repeated’ episodes of absence were defined as two or more episodes of absence. Transferral was defined as having had unplanned changes of ward of training due to: failure to thrive or due to inadequate development of competences. Residents were considered to be dropouts if they were dismissed from, had resigned from, or changed their speciality. Exclusion criteria were: decelerations due to maternity leave, including unknown absence three months before/after maternity leave, as well as changes of specialty during the introductory training positions (non-residency positions), which were not uncommon events. Controls were a random sample of doctors in the source population, who were not identified as cases in the case extraction period. We aimed at a case–control ratio of 1:4 and matched cases and controls on graduation year.

### Data extraction

Cases were identified after evaluation of individual residency records in an existing database (evaluer.dk), which tracks the progression of individual doctors in postgraduate training in Denmark, and via the records of the regional Council for Postgraduate Medical Training. Lists of all potential controls (graduates from the same year and institution) were supplied by administrators at Aarhus University (AU). The order of graduates in each cohort of potential controls was randomized by researchers using the website random.org. Subsequently, potential controls were checked for membership and source population and picked in this random order if they belonged to the source population. Data on medical school performance and progression was extracted from the AU administrative databases, and data on dispensations (dates and causes for seeking academic dispensations) were extracted manually from paper files in a physical archive at AU. Data extraction took place between June 2013 and February 2014.

### Ethics

The study was exempt from ethics review by the regional ethics committee. The authors also obtained permission from the Danish Data Protection Agency to use and combine the specific data extracted from the specific sources for the purpose of this study as required by Danish law.

### Variables

Potential predictor variables representing available measures of medical school performances relating to re-examinations, grades, completion time, and dispensations were examined, as was demographic variables.

### Demographics

A variable for gender was supplied with the data set from AU. We formed a variable representing the age at admission to medical school (“Admission age”), by subtracting students’ birth date from the date they were registered as starting medical school at AU.

### Re-examinations

Three variables were formed representing the number of re-examinations in medical school’ taken by students during the first year (“‘Year 1 re-examinations”), the first two years (“Year 1 & 2 re-examinations”), and in total (“Total re-examinations”).

### Medical school grades

Medical school grades were calculated as an equally weighted average of all number grades given to a student during medical school, i.e. pass/fail grades were excluded. The grading scale was the Danish seven-point grades scale that contains the grades: −3 (unacceptable), 0 (inadequate), 2 (adequate), 4 (fair), 7 (good), 10 (very good), and 12 (excellent).

### Completion time

Programme completion time (“Completion time”) was calculated as the number of years between the day students commenced the medical programme administratively and the day of the last examination. The current rules of progression allow students up to 12 years to complete the six-year medical school curriculum.

### Dispensations

Students had to seek dispensations if their progression deviated from academic rules and regulations, i.e. if they required extra time for examinations for some reason, or if they needed more than three attempts to pass an examination, etc. We generated one variable for the number of dispensations sought during the first year of study (“Year 1 dispensations”) and one for the total number of dispensations sought in medical school (“Total dispensations”). In addition we categorized the causes given for seeking dispensations into a number of relevant categories. The categorization was performed independently by two researchers.

### Analysis

Differences amongst cases and controls were examined with either t-tests (continuous data), or Χ^2^ tests or Fisher’s exact tests (categorical data). We used conditional logistic regression to examine predictor variables of case-status and to estimate odds ratios (OR) and their 95% confidence intervals (95% CI). Before multivariate analysis all predictor variables were screened for collinearity and zero cells. We examined a multivariate model using backwards hierarchical elimination [19], and one in which we included only the significant (p < 0.05) univariate predictor variables. Descriptive summary statistics and analyses were performed using STATA/IC 12.

## Results

We identified 89 cases and 343 controls in the source population. Of the 89 cases 40 decelerated, 35 dropped out, 3 transferred and 11 were combinations of these outcomes. The academic dispensation applications for cases and controls could be categorized into eight overall categories, which are summarized in Table 1. These eight categories covered all dispensation applications encountered (Table 1). There was agreement between the two researchers about the categorization of dispensation applications in more than 90% of cases the first time round. Cases and controls are summarized on the variables of interest in Table 2. As seen in Table 2, cases had significantly more examination attempts (p = 0.036) and took significantly longer time to complete medical school (p = 0.013) compared to controls. However, the final competency levels as expressed by the average medical school grades were not significantly different for cases and controls by the end of medical school (p = 0.246).

**Table 1:** **Causes for seeking academic dispensations in cases and controls (n = 432)**

**Category name**	**Description**
Poor organizational skills	Applications for dispensations due to missed deadlines, e.g. late registrations for exams or submission of assignments, etc.
Pregnancy	Applications for extended examination time, changes in order and timing of courses and examinations, etc. due to pregnancy.
Family problems	Applications grounded in problematic or serious familial events or situations, e.g. burials, parental responsibilities, life threatening disease, etc.
Impairment	Applications—typically relating to examinations (i.e. extended examination time, extra exam attempts, re-examinations)—which were caused by chronic illnesses, handicaps, or dyslexia.
Academic problems	Applications for a 4th or 5th exam attempt where no causes were given, as well as applications for remediation activities.
Illness	Applications for re-examinations on grounds of shorter lasting illnesses such as influenza, etc.
Study plan deviations	Applications for changes from the standard study plan, i.e. changes in the order of subjects, time of examination, etc. We distinguished between three subcategories of study plan deviations: positive, neutral or negative. In the positive subcategory the causes related to relevant research or international exchange activities, etc. In the neutral category we were either unable to ascertain a cause or unable to determine whether a cause was positive or negative. Finally, in the negative deviations category, there were clear signs of students prioritizing other activities above medical school activities, for example: holidays booked during terms, family birthdays, sport activities, etc., or signs that students were struggling to keep up with the ordinary study plan.
Merits	Applications for merits for courses taken in other programs, or advance approval of planned relevant educational activities such as electives and placements, etc.

**Table 2:** **Summary of medical school performance indicators for cases (n = 89) and controls (n = 343)**

**Variable**	**Residents in difficulty n**	**Controls n**	**p-value**
Gender			
- Female	59	208	0.956
- Male	30	135	
Admission age (years)	89	343	
	Mean 21.94	Mean 21.67	0.270
	SD 1.74	SD 2.13	
Graduation year (calendar year)	89	343	
	Median 2004	Median 2004	0.322
	Range 1978–2011	Range 1978–2011	
Year 1 re-examinations	89	343	
	Mean 0.11	Mean 0.15	0.457
	SD 0.35	SD 0.42	
Year 1 & 2 re-examinations	89	343	
	Mean 0.45	Mean 0.43	0.832
	SD 0.71	SD 0.85	
Total re-examinations	89	343	
	Mean 3.01	Mean 2.01	0.032
	SD 4.71	SD 3.28	
Medical school grades (Danish 7-point scale)	89	343	
	Mean 7.17	Mean 7.34	0.320
	SD 1.44	SD 1.39	
	Range 4.89–10.30	Range 3.88–11.13	
Completion time (years)	89	343	
	Mean 7.56	Mean 7.19	0.013
	SD 1.33	SD 1.19	
Year 1 dispensations	89	343	
	Mean 0.04	Mean 0.05	0.883
	SD 0.03	SD 0.01	
Total dispensations	89	89	
	Mean 0.44	Mean 0.34	0.362
	SD 0.95	SD 0.95	
Poor organizational skills			
- No	89	333	0.097
- Yes	0	10	
Pregnancy			
- No	85	324	0.469
- Yes	4	19	
Family problems			
- No	86	339	0.157
- Yes	3	4	
Impairment			
- No	86	338	0.215
- Yes	3	5	
Academic problems			
- No	87	336	0.584
- Yes	2	7	
Illness			
- No	85	329	0.527
- Yes	4	14	
Study plan deviations			
- None	82	328	0.497
- Positive	3	5	
- Neutral	3	6	
- Negative	1	4	
Merits			
- No	83	328	0.249
- Yes	6	15	

The total number of re-examinations during medical school and the time it took to complete medical school were the only significant univariate predictors of being a resident experiencing deceleration, transferral or dropout (Table 3). Subgroup analyses revealed that the association with completion time was strongest for those 35 residents in difficulty who dropped out of residency. Neither admission age, gender, medical school grades, nor the total number of academic dispensation applications in medical school were significant univariate predictors of being a resident in difficulty (Table 3). In addition, none of the indicators of early medical school performance (year 1–2 re-examinations or dispensations) were associated with subsequent difficulty in residency. Because so few cases and controls in general sought academic dispensations during medical school, the eight categories of academic dispensation resulted in very small cells (Table 2), a situation which may result in biased estimates when examining prediction with conditional logistic regression analyses [20]; therefore, these variables were not included in the regression analyses (Table 3). Model 1 in Table 3 is a multivariate model resulting from a backwards elimination process of a starting model containing all nine univariate predictors. As seen in model 1, only completion time survives as an independent significant predictor of case status. We also tested model 2 containing the only two significant univariate predictor variables (total re-examinations and completion time); however, this model was not stable and not a better model of prediction than the univariate predictors on their own (Table 3). We found that the total number of re-examinations and the time it took to complete medical school were correlated (r = 0.432, p = 0.000).

**Table 3:** **Predictors of struggling in residency**

	**Univariate analyses**		**Model 1**		**Model 2**	
	OR (95% CI)	p-value	OR (95% CI)	p-value	OR (95% CI)	p-value
Male gender	0.77 (0.47–1.27)	0.306				
Admission age	1.07 (0.96–1.19)	0.203	1.09 (0.98–1.22)	0.121		
Year 1 re-examinations	0.78 (0.42–1.47)	0.442				
Year 1 & 2 re-examinations	1.02 (0.77–1.36)	0.866				
Total no. of re-examinations	1.06 (1.00–1.13)	0.036			1.03 (0.97–1.11)	0.313
Average medical school grades	0.90 (0.76–1.07)	0.246				
Completion time (years)	1.28 (1.05–1.57)	0.016	1.30 (1.06–1.58)	0.011	1.21 (0.96–1.52)	0.102
No. of year 1 dispensations	0.95 (0.38–2.39)	0.913				
Total no. of dispensations	1.12 (0.89–1.41)	0.324				
n_obs_	432		432		432	
LR Χ^2^			8.29		7.00	
LR p-value			0.0158		0.0302	
Pseudo R^2^			0.0215		0.0181	

OR is the odds ratio; 95% CI is the 95% confidence interval; n_obs_ = the number of cases and controls used included in the analysis; LR = Likelihood ratio test. The differences in p-values observed in Tables 2 and 3 for the same variables are a result of using different statistics, i.e. t-tests, Χ^2^ test or Fisher’s exact test were used in Table 2 and conditional logistic regression statistics were used in Table 3.

For an increase in time to complete medical school of one year, the odds of ending up as a resident in difficulty increased 28% (OR = 1.28; Table 4). The odds of ending up being a resident in difficulty were more than twice as large for a student who finished medical school nine years from study start (+3 years) compared to students who finished on schedule, i.e. after six years (OR = 2.12; Table 4). Similarly, if we look at re-examinations in isolation, the odds of being a resident in difficulty were 44% larger for students with a history of six failed exam attempts in medical school compared to a student who passed all exams in the first attempt (Table 4).

**Table 4:** **Exposures and odds ratios for struggling in residency**

**Exposure**	**Unadjusted OR (95% CI)**
Medical school completion time:	
+1 year	1.28 (1.05–1.57)
+2 years	1.65 (1.10–2.48)
+3 years	2.12 (1.15–3.90)
+5 years	3.50 (1.26–9.67)
+10 years	12.23 (1.60–93.48)
No. of medical school re-examinations:	
2	1.13 (1.01–1.27)
4	1.28 (1.02–1.61)
6	1.44 (1.03–2.04)
8	1.63 (1.03–2.58)
10	1.85 (1.04–3.27)

OR = Unadjusted odds ratios; 95% CI = 95% confidence intervals for the OR.

## Discussion

We found the total number of re-examinations during medical school and the time it took to complete medical school to be significant univariate predictors of observable difficulties in residency training (deceleration, transferral or dropout). We were not able to model case status with a multivariate model when using backwards hierarchical elimination. We suspect that the bivariate model (model 2) that included both of the significant univariate predictors (total re-examinations and completion) were unstable due to collinearity, as the two predictors were correlated (r = 0.432, p = 0.000), because repeated examination failures generally tend to mess up students’ study plans and delay their completion. Another cause of late completion may be leaves of absence during medical school, which has previously been shown to be associated with drop out from this particular medical programme [17]. It is therefore likely that late completion may be associated not only with academic problems like failed examinations, but perhaps also with factors such as stress/burnout, or perhaps with a waning commitment to medicine. One review of the literature on residents in difficulty found that they were most often identified by programme directors on the grounds of insufficient medical knowledge and poor clinical judgment, but also on inefficient use of time [7]. Other authors have reported absenteeism, poor attendance, being slow or late as observable behaviours of both struggling medical students and residents in difficulty [3,6,21-24]. Our results suggest that both prior academic challenges (repeated exam failures) and being a slow learner (longer completion times) were associated with struggling to comply with the demands of subsequent residency training. In contrast to recent meta-analyses and reviews [8-11], we did not find any association between medical school grades and residency performances (Table 3). There is certainly a universal tendency for “restriction of range” when using medical school grades as predictors of performance in postgraduate training, which may diminish the chance of finding strong associations.^8^ Restriction of range probably also affected our results, since the academically worst performing medical students at AU tend to drop out early in medical school, and the overall dropout rate has been found to be quite high (20%) [24,25]. While correction for restriction of range is statistically possible [8], we do not find it valid considering the aim of this study (early identification), as residents do no present themselves in the wards with academic records, which are corrected for unreliability or restriction of range. Another, and most likely better explanation for the lack of predictive power of medical school grades in this context, is the fact that Danish universities are bound by national laws to be very liberal with examination attempts (minimum three per subject) and completion times (maximum 12 years for a six-year programme). *That is given some extra learning time and examination attempts, our cases eventually reached similar levels of competence in medical school as controls* (Table 2). In addition, we did not find significant differences in the patterns of dispensation applications amongst our cases and controls either (Tables 2), which we would expect if cases were considerably weaker than controls academically. Local researchers recently examined dispensation applications in this same medical programme with a retrospective cohort study of 1,056 medical students admitted between 2003 and 2006. A significantly larger proportion of dispensations seekers dropped out compared to non-dispensations seekers (58% versus 17.6% dropout) [25]. It is therefore likely that students with the most severe academic problems reflected in academic dispensations never made it through to graduation, residency or to our sample.

Our results would suggest that ignoring past performances is not the best way to help at-risk doctors with a smooth transition to postgraduate training. Instead, specific medical school performance indicators could be included in the introductory dialogues between doctors and workplaces in order to align expectations before designing and implementing tailored, individual learning plans. Our results would suggest that residents in difficulty were slower learners, and they could be under time pressure in residency programmes because of the fixed-length employment relationships. They may struggle because fixed-length training programmes are too rigid for “slow learners”—not necessarily because they will become poorer doctors long term. In other words, the “one-size-fits-all” approach to residency training could be seen as the main problem, and the proven learning potential of residents in difficulty as the solution. Recent developments in competency-based education have highlighted the need for a shift from “fixed-length variable-outcome programmes” to “fixed-outcome variable-length programmes” [26-30]. Ten Cate *et al*. emphasized that “the goal of competency-based education should be linked to observed competence, rather than to the current proxy measure, length of training”, and that “as educators, we should recognize that all trainees do not reach the same level of competence for all activities at exactly the same time” [29]. Our results seem to support that line of thinking, because while there were no significant differences in the final average medical school grades or “competences” of cases and controls, there were differences in the “process” of reaching similar competency levels.

### Strengths and limitations

The strength of this case–control study was that there was no missing data in the data extracted from the database, and that recall bias was not an issue. On the other hand, the databases and archives from which data was extracted, were kept and maintained for administrative purposes, and they may have contained errors and have been incomplete (e.g. missing student dispensation case paper files or missing documents within these files). We had no control over these types of bias with this design. Misclassification of variables is also always a possibility when handling large amounts of complex data as in this case. The collinearity mentioned above is a likely limitation, which may have prevented the control for confounding. A methodological limitation is that matching for graduation year does not secure the exact same exposures during medical school for cases and controls, since completion time does vary; however, there is no immediate solution to that problem [5]. It was a strength that we were able to examine predictors of difficulties in residency across all types of specialties in two of five Danish regions. On the other hand, it was a limitation that we only had access to medical school performance data for doctors graduating from one particular institution. Finally, it is a strength that we are able to report results from a different educational and cultural setting than most of the previously published literature on the subject [8-11]—a setting with liberal progression rules for pre-graduate medical education.

## Conclusions

Residents in difficulty eventually reached similar competence levels as controls during medical school when given the opportunity. However, they needed more exam attempts and longer time to complete their pre-graduate studies, and so seemed to be slower learners in general. Knowledge of relevant medical school performance indicators could be used constructively in discussions with the resident to tailor and plan a more appropriate and individual residency. A change from “fixed-length variable-outcome programmes” to “fixed-outcome variable-length programmes” has been proposed as a way of dealing with the fact that not all learners reach the same level of competence for all activities at exactly the same time. This study seems to support the logic of such an approach to residents in difficulty.
